# Meiosis in a Bottle: New Approaches to Overcome Mammalian Meiocyte Study Limitations

**DOI:** 10.3390/genes2010152

**Published:** 2011-02-12

**Authors:** Ignasi Roig, Miguel Angel Brieno-Enriquez, Montserrat Garcia Caldes

**Affiliations:** 1 Cytology and Histology Unit, Cell Biology, Physiology and Immunology Department, Universitat Autònoma de Barcelona, 08193 Cerdanyola del Vallès, Barcelona, Spain; 2 Cell Biology and Medical Genetics Unit, Cell Biology, Physiology and Immunology Department, Universitat Autònoma de Barcelona, 08193 Cerdanyola del Vallès, Barcelona, Spain; E-Mail: mikelenriquez@hotmail.com; 3 Farmacology and Physiology Department, School of Medicine, Universidad Autónoma de San Luis Potosí, San Luis Potosí, Mexico

**Keywords:** meiosis, *in vitro*, mammals

## Abstract

The study of meiosis is limited because of the intrinsic nature of gametogenesis in mammals. One way to overcome these limitations would be the use of culture systems that would allow meiotic progression *in vitro*. There have been some attempts to culture mammalian meiocytes in recent years. In this review we will summarize all the efforts to-date in order to culture mammalian sperm and oocyte precursor cells.

## 1. Introduction

Gametogenesis is the process by which gametes are produced. During this process, haploid cells are obtained from diploid progenitors. This is achieved by executing the meiotic program which involves two successive rounds of cell division after a single round of DNA replication. The proper execution of this program is essential to maintain species ploidy from one generation to the next, as well as to provide genetic variation to the species. Thus, errors produced during meiosis can compromise fertility or cause aneuploid embryos. In humans, the impact of errors produced during meiotic divisions is an important social problem. Aneuploidy is the major cause of mental retardation and developmental issues present in newborns [1]. Moreover, it is believed that most miscarried conceptions do not come to term because of the presence of an unbalanced karyotype [1]. Therefore, to understand the origin of aneuploidy and infertility, it is crucial to know in detail the processes that happen during meiosis and gametogenesis.

In mammals, gametogenesis is initiated early in development. In mice, around embryonic day (E)7.25, specification of germ cells occurs. During this process, a group of cells from the most proximal epiblast is induced to differentiate into primordial germ cells (PGCs) [2,3]. These newly formed PGCs will proliferate and migrate to the embryonic gonads, where they can be found around E10.5. Once in the gonads, PGCs undergo several rounds of mitosis. By E12.5, PGCs express some meiotic-specific genes, such as *Sycp3* and *Dmc1* [4]. Until this point this process is sex-independent. Then, in male gonads, germ cells downregulate meiotic-specific genes and initiate a mitotic arrest [5]. In contrast, in the female gonads, germ cells enter meiosis, complete meiotic prophase and arrest at dictyate stage at least until puberty begins. Meiotic entrance is regulated by the presence of intrinsic factors like *Dazl*, [6] and also by extrinsic ones like retinoic acid (RA) [7,8]. Fetal female gonads have much higher concentration of RA than male ones, most likely because male gonads express the enzyme responsible for catalysis of RA, CYP25B1 [7]. Moreover, *in vitro* studies have shown that addition of RA to culture media induces meiosis initiation in male embryonic gonads *in vitro* [7,8].

Later on, in the adult life of mammalian species, gender-related differences regarding evolution of meiosis can be found. While females only have a limited pool of arrested meiotic cells available to complete meiosis during their reproductive lifespan, males have an almost unlimited capability to create spermatocytes, thus allowing males to have a significantly longer fertility than females. In adult males, arrested cells resume mitotic activity, produce spermatogonia which constantly enter meiosis to produce sperm during most of the life of the individual.

The first knowledge about mammalian meiosis arose from the descriptive analysis of different species gonads, like rodents (especially mouse and rat), some domestic animals (pig, dog, cattle, *etc.*) and primates, especially humans. With the production of genetically engineered mice and their use to study the role of different genes in mouse meiosis, a more detailed image of the mechanisms regulating meiosis and gametogenesis have been drawn. Nevertheless, in most cases, and because of the intrinsic nature of female gametogenesis, most studies are performed using male samples. It is important to notice that male and female gametogenesis, and meiosis in particular, does not respond the same way to the same perturbation [9,10]. Therefore, analysis should be carried out in both male and female gonads.

While studies of genetically engineered mice have provided a wealth of information regarding many molecular pathways, there are technical limitations imposed by this approach. Some of the more important are that the production of genetic engineered mice needs time and substantial economical efforts. Thus, it would be very valuable to have a tool to predict the outcome of such an expensive experiment. Moreover, having an *in vitro* culture that could allow meiosis *in vitro* would certainly reduce the number of experimental animals needed to be used in future studies. In addition, it would allow a more detailed analysis of the human meiotic process to try to address human infertility and aneuploidy origin directly. An additional advantage of *in vitro* approaches is that they allow for manipulation of meiotic events such as RNA interference and introduction of environmental toxins, as just two examples. In this sense, some efforts have been focused on promoting entrance of cells into the meiotic program or to culture meiocytes *in vitro*. In this review we will summarize all the efforts carried out until now to study meiosis *in vitro* and analyze the perspective of this new exiting approach for the study of mammalian meiosis.

## 2. Stem Cells Culture to Obtain Sperm-Like and Oocyte-Like Cells

One of the strategies to obtain meiotic cultures arises from the knowledge that germ cells can spontaneously originate from embryonic stem (ES) cells in culture [11–14]. ES cells (ESCs) are derived from the inner cell mass of the preimplantation embryo and maintain pluripotency, defined as the capacity to develop into any cell type of somatic ectodermal, mesodermal, or endodermal lineages. ESCs can also develop into the germline, as shown by mouse blastocyst injection [15] and by *in vitro* differentiation of mouse and human ESCs (see below). Several reports have recently documented primordial germ cell- (PGC), sperm-, and oocyte-like cell development after mouse and human ESC differentiation (see below).

Several groups noticed that ES cells in culture can differentiate into germ cells precursors. This process occurs at a very low rate if no stimuli are applied. To improve it, some groups have now developed different strategies to promote differentiation of mouse ES cells into germ cells, some of them obtaining haploid cells able to produce progeny.

The first approach to obtain mammalian germ cells was done by Toyooka and colleagues [13]. Because it has been shown that bone morphogenic proteins (BMPs) have a crucial role in promoting germ cell differentiation *in vivo* [16,17], these authors co-cultured ESCs with M15 or trophoblast cells that produce high levels of BMPs to induce ESC differentiation into PGCs *in vitro*. ESCs were genetically modified and contained either GFP or the LacZ gene in the endogenous *Vasa* loci, which allowed PGC detection. First signs of differentiation were detectable after one day of co-culture. Exposure to BMPs allowed the recovery of almost 300 times more PGCs than in unexposed ESC cultures. These knock-in PGCs were transplanted into host testis and eight weeks after transplant sperm sharing the same markers than the transplanted PGCs were found [13]. Nevertheless, no functionality test of the obtained sperm was performed. More recently, other studies have demonstrated that BMPs can also induce PGC differentiation in human ESCs [18].

Geijsen and colleagues [11] used RA to induce ESC differentiation into PGCs. In this case, *in vitro* differentiation did not stop at PGCs and haploid cells were detectable after culture. Although meiotic progression was highly inefficient in this culture setting, this study represents the first one that obtained haploid cells from mouse ESCs *in vitro*. Moreover, 20% of the haploid cells used in a test to *in vitro* fertilize oocytes formed embryos that progressed to blastocyst stage [11]. Other groups have used similar approaches using RA to generate haploid cells from mouse ESCs [12]. In this study, approximately 30% of the cells in culture after 72 h of RA induction were haploid. Furthermore, 12 animals were born after intracytoplasmic injection of the *in vitro*-generated haploid cells into unfertilized oocytes and transfer to pseudo-pregnant females (*N* = 65). Nevertheless, the offspring died prematurely, probably due to imprinting problems of the male haploid cells.

Another study has reported the derivation of germ cells from bone marrow stem cells [19]. In this case, RA was used as an inducing agent to obtain PGC-like cells. However, these cells were unable to restore spermatogenesis when transplanted to germ cell-depleted testis.

More recently, the first report of differentiation of male haploid germ cells from human ES cells has been published [20]. In this study, the authors purified PGC-like cells from human ES cells and induced their differentiation to meiotic and post-meiotic male germ cells by overexpressing DAZ gene family members: *DAZL*, *BOULE* and *DAZ*. Results show that after 14 days of culture, *TEKT1*, a marker of mature sperm, can be detected by RT-PCR. Moreover, approximately 2% of the cells in culture are haploid. This study represents the first demonstration that human germ cells can be also differentiated from pluripotent ES cells *in vitro*.

Hubner *et al.* [21] reported the differentiation of mouse ESCs into PGCs, these cells were transfected with a GFP reporter under the control of the germ cell-specific promoter *Oct4*. GFP was expressed in undifferentiated ESCs but was expected to disappear in differentiated cells, these authors also analyzed different markers of differentiation like *c-KIT*, *Vasa* expression and c-KIT−/GFP+ postmigratory germ cells with high *Vasa* expression. Germ cells that down-regulated OCT4 expression entered meiotic prophase. During days 12 to 26, the floating ESC-derived aggregates developed into morphologically visible follicle-like structures that contained putative oocytes. These follicular structures also expressed *Gdf9*, steroidogenic enzymes, and produced estrogen. On day 26, the follicles released oocytes of 50–70 μm in diameter that expressed the oocyte specific markers *Zp2/3* and *Figla*. Furthermore, pre-implantation stage embryos were observed and were likely the result of parthenogenetic oocyte activation. Notably, both XX and XY mouse ESC lines produced oocyte-like cells. Nuclear SYCP3 was detected in ESC-derived oocyte-like cells; however, no pairing-synapsis was detected.

Subsequently, Novak *et al.* [23], performing a protocol similar to the one described by Hubner and colleagues [21], observed the presence of follicle-like aggregates and elevated levels of estrogens in the supernatant after 12 days of culture of mouse ESCs. Differentiation to oocyte-like cells of 40% was observed between 14 and 16 days of culture. The differentiation consisted of cells being SYCP3 positive; however, nuclear localization was variable. Moreover, instead of the long chromosome axial core alignment of SYCP3, only short filamentous structures were observed in the aggregates, suggesting an abnormal loading of SCP3. The expression of meiotic genes like *Dmc1*, *Sycp1* and *Sycp2*, was not detected, and synapsis was disrupted. In both cases [21], the ESC-derived oocyte-like cells produced were not able to progress into meiosis.

Similarly, Lacham-Kaplan [22] found differentiation for two to three weeks in testicular conditioned media resulted in an increased number and size of follicle-like structures [22,23]. These follicle-like cell clusters contained putative oocyte-like cells of up to 35 μm in diameter and expressed markers of oogenesis, like *Stra8*, *Figla*, and *Zp3*. However, mouse ESC-derived oocyte-like cell maturation, oocyte functionality or their ability to be fertilized and produce offspring was not demonstrated. Furthermore, meiotic progression of ESC-derived oocyte-like cells was not assessed.

Recently, Qing *et al.* [24], using a two-step method, induced the differentiation of mouse ESCs into oocyte-like cells [24]. Under certain *in vitro* conditions, ESCs can aggregate and grow, forming a colony known as an embryoid body (EB). In Qing *et al.* [24], PGCs were differentiated within the EB around day 4 of culture. After that, EBs were co-cultured with ovarian granulosa cells. After 10 days, these cells formed germ cell colonies as indicated by the expression of the two germ cell markers *Vasa* and *Sycp3*. These cells also expressed oocyte-specific genes (e.g., *FIGalpha*, *GDF-9*, and *Zp1-3*) but not any testis-specific genes. EB cultured alone, or cultured in granulosa cell-conditioned medium or EB co-cultured with Chinese hamster ovary (CHO) cells, or cultured in CHO cell-conditioned medium did not express any of these oocyte-specific markers. Immunocytochemistry analysis using antibodies against VASA or GDF-9 confirmed that some double-positive oocyte-like cells were generated within the germ cell colonies. These results demonstrate that granulosa cells were effective in inducing the differentiation of ESC-derived PGCs into oocyte-like cells most likely through direct cell-to-cell contacts.

Kerkis *et al.* [25] used a RA-induced differentiation protocol to produce sperm-like and oocyte-like cells from male mouse ESCs. In different experiments they generated male or female gametes, using genetically manipulated or preselected ES cells. XY mouse ESCs were differentiated in suspension as EBs for 4 days without RA and 4 more days with 0.1 μm RA. Cells on the periphery of the EBs appeared to have a different morphology and express germ cell markers including *Ssea1*, *Oct4*, *Dazl*, *Vasa*, *Stra8*, *Sycp1*, *Sycp3*, and *Zp3*. Although these transcripts were also expressed in undifferentiated ESCs, the expression of *Gdf9* and *Acrosin* was low to absent in ESCs and substantially increased during differentiation. By cytogenetic analysis, they observed a chromosome reduction in ES-derived GC. Finally, they concluded that ESCs with XY chromosomes can produce both types of gametes under the same experimental conditions [25]. Differentiation will depend on their positional and temporal information within the EB context.

Finally, Salvador *et al.* [26], using GFP as a marker under the regulation of the *Gdf9* promoter, reported the identification of oocyte-like cells in cultures of XX mouse ESCs [26]. After differentiation for a day on feeders or in suspension without leukemia inhibitory factor (LIF), GFP positive oocyte-like cells were detected in the supernatant. These cells expressed *Gdf9* and *Zp3*. Surprisingly, the addition of LIF to the culture media increased the number of GFP positive cells by three-fold. However, follicle-like structures were not detected, and the oocyte-like cells quickly degenerated, which the authors related to the inability of the ESC-derived oocytes to properly execute meiosis.

Many challenges need to be overcome to achieve robust and functional gamete differentiation from ESCs. Particularly, the low efficiencies of ESC-derived oocyte-like cell maturation reported to date may be unavoidable because naturally in mammals most oocytes undergo atresia during fetal development. However, the optimization of methods to direct ESC-derived germ cell specification, oocyte commitment, and oocyte maturation may increase efficiencies and enable functional oocyte production.

## 3. Culture of Adult Male Germ Cells

Cultures of mammalian male germ cells are rare and not many reports have been published. Nevertheless, the few published studies succeeded in promoting meiotic progression *in vitro* and obtaining haploid cells.

Some of the first attempts to culture mammalian male germ cells were carried out by Durand and colleagues over the past decade [27–31]. Over this time they have developed a co-culture technique that allows meiotic progression of rat spermatocytes. Briefly, it consists of co-culture of prepuberal rat spermatocytes on a monolayer of Sertoli cells in a bicameral chamber [31]. In their experiments, meiotic progression was followed by different approaches, including electron microscopy ultrastructural analysis of the cultured cells, analysis of the DNA content by fluorescence activated cell sorting (FACS) and expression of some postmeiotic specific genes. All analysis performed suggested the existence of meiotic progression *in vitro* achieving haploid cells after three weeks of culture [27]. Moreover, when the culture was seeded with BrdU-labeled leptotene-stage spermatocytes, BrdU-positive round spermatids were observed after 21 days of culture [30]. More importantly, evolution of spermatogenesis *in vitro* followed similar dynamics to what happened *in vivo* [28] Nevertheless, the functionality of the haploid cells has never been tested.

Another approach to culture male germ cell was described by Feng and colleagues [32], who purified undifferentiated type A spermatogonia from 6 days postpartum mice and immortalized them by overexpressing TERT. Then, they induced their differentiation by culturing them in the presence of stem cell factor (SCF), which is known to play a crucial role in the initiation of spermatogenesis. After a week in culture, there was a seven-fold increase in the number of cells with four sets of chromosomes, presumably indicating an increase in the number of primary spermatocytes. During culture, SYCP3 positive cells were evident and chismata could be observed in Giemsa-stained cells. After two weeks in culture, expression of postmeiotic markers, like *SP-10* and *protamine-2*, were observed. Approximately 60% of the cells in culture were haploid after three weeks of SCF induction. Nevertheless, authors never observed any structure resembling sperm tails nor did they test the competence of the haploid cells obtained in culture [32].

Finally, another culture system has been developed to cultivate testis cells from non-obstructive azoospermic *in vitro* fertilization clinic patients [33]. Testis biopsies were dissociated and the obtained cells were cultured in a collagen matrix for 12 days. Analysis after this time revealed a decrease in the number of pachytene-stage spermatocytes as well as an increase in the number of spermatids. Thus, authors suggest that human haploid cells can be formed *in vitro*, but again more experiments should be performed to check if these spermatid-like cells are able to fertilize oocytes [33].

Culture of adult spermatocyte cell precursors is nowadays close to being a reality. Obtaining haploid cells from diploid progenitors represents complete meiotic progression *in vitro*. Therefore, it is only a matter of time until more refined techniques will be available to address some key issues about meiosis. In fact, some studies are already using cultures of spermatogenic cells to test toxicity of certain drugs or agents on mammalian gonads [29,34].

## 4. Attempts to Culture Mammalian Fetal Oocytes

The culture of human fetal oocytes has been tried by a few researchers and a great variety of techniques as well as a variety of culture media has been evaluated. The oldest reports are from Blandau [35] who reported the presence of living oogonia and oocytes for almost 80 days [35]. During culturing, he observed active migration of oogonia, mitotic divisions and growth of the fetal tissue. He concluded that oogonia entered meiosis, and generated oocytes that reached late stages of meiosis. Later reports [36] analyzed the progression in culture of human ovarian fragments from fetuses of 16–20 weeks of gestation. Ovaries used were from fresh as well as frozen samples. Results, published by Zhang and colleagues included the persistence of primordial follicles in culture and oocytes that had extruded a polar body.

More recently reports from [37] evaluated human fetal ovaries aged 13–16 weeks. Ovaries were cultured in mini-blocks (0.3 × 0.3 × 0.3 mm) with minimal essential medium alpha (MEMα) supplemented with fetal calf serum (FCS) and follicle stimulating hormone. Another two supplements were added depending on the group (human female serum and FCS suitable for stem cells). The cultures were analyzed after 7 to 40 days. These authors observed that the number of oocytes and the percentages of zygotene stage and pachytene stage cells increased with the time of culture. The elevated initial number of degenerated cells decreased with the time of culture. Oocytes cultured with human female serum and FCS for stem cells showed a higher number of oocytes after 14 days of culture. Cultured oocytes showed the presence of lateral element of synaptonemal complex, indicating meiotic progression. The analysis of the formation of the synaptonemal complex showed a high index of asynaptic nucleus, self synapsis and non-homologous synapsis. The authors concluded that human oocytes survive in culture, and more importantly, can progress through prophase I *in vitro*.

A few years later, the first mouse fetal ovary culture was described [38]. One hundred and sixty-two ovaries from 13, 14 or 17 days post-coitum embryos were cultured in αMEM supplemented with fetal calf serum for stem cells (ES-FCS), penicillin, and streptomycin. This media was used for half of the cultures; the other half was cultured with the addition of stem cell factor (SCF), insulin growth factor I and LIF. The cultures were analyzed for meiotic pairing-synapsis and recombination after three or four days. Authors reported a significant increase in the number of oocytes that reached pachynema after four days of culture in those supplemented cultures. However, none of the cultured oocytes displayed MLH1 (a cytological marker for crossover formation which appears around pachynema). Nevertheless, authors concluded that supplementation with SCF, LIF and IGF-I promotes female meiosis *in vitro*, and proposed that this increment in cells at pachytene stage could be related to an increment of premeiotic oogonias that entered meiosis because of the growth factor stimuli.

Roig *et al.* [39] described another culture approach to promote oocyte I progression *in vitro*. Ovaries were cut in blocks (approximately 15 × 20 × 20 mm) and cultured in αMEM, supplemented with human albumin, insulin, transferrin, selenium, penicillin and streptomycin for one to five weeks. Analysis of meiotic progression focused on homologous pairing as well as synapsis progression. This study demonstrated that human fetal oocytes could survive *in vitro* up to five weeks [39]. In three of the four cases they observed meiotic progression. Although the percentages of oocytes at the different stages of meiosis as well as the proportions among the different cases were different from the ones observed in fresh oocytes, pairing and synapsis efficiency was similar to those described in fresh oocytes. Finally, these authors also described an increment of degenerated cells in relation with the time of culture.

More recently another report has been published in which the effects of different culture approaches are compared in order to culture human fetal oocytes [40]. Authors reported that disaggregation by mechanical methods increased the total number of oocytes found in culture, but also decreased the number of degenerated cells. Similarly, oocytes cultured with SCF, independently of the disaggregation method used, showed a higher proportion of oocytes that reached pachynema and decreased the number of degenerated cells. This study also evaluated meiotic recombination in oocytes from ovaries disaggregated with mechanical methods and cultured with SCF. The ranges of MLH1 foci found in cultured oocytes, as well as the means, mimic the values reported in fresh oocytes. Therefore, authors concluded that oocytes cultured with this method resembled, for the studied parameters, the oocytes found *in vivo*.

In summary, all the published reports to-date present a promising future in order to culture fetal oocytes. It is now clear that oocytes can be maintained and progress *in vitro*, but more interestingly, that meiosis can be initiated in culture.

## 5. Follicle and Oocyte Growth and Development in Culture

**Figure 1:**
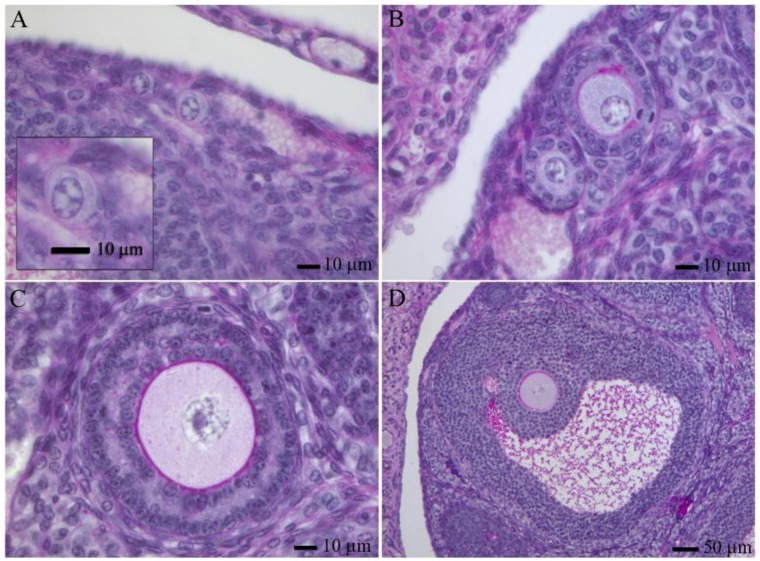
Follicle development in the adult mouse ovary. Primordial (**A**), primary (**B**), pre-antral (**C**) and antral (**D**) follicles stained with PAS-hematoxilin [42].

Adult mammalian ovaries contain follicles, which are the structures in which a post-prophase I oocyte develops. They consist of the oocyte themselves, surrounded by granulose and theca cells. In the adult ovary, one can find follicles at different developmental stages, the most abundant are primordial follicles. Based in morphology, human follicles can be classified (Figure 1, reviewed by [41]). Human follicles can be classified as primordial follicles (those that contain a primary oocyte embraced by flattened granulosa cells, Figure 1A), primary follicles (have a full cuboidal granulosa cell layer, Figure 1B), preantral follicles (growing primary oocytes enclosed by several granulosa cell layers, Figure 1C) and antral follicles (which contain a cavity with follicular fluid, granulosa cells differentiate into mural and cumulus cells, Figure 1D). Except for the oocytes from antral follicles, that usually need a short period of manipulation and maturation in culture, oocytes from small antral follicles and pre-antral follicles require long periods of growth *in vitro* to acquire developmental competence. There are many publications related to this field, in this sense we will only mention some of the most representative ones.

The success of *in vitro* growth (IVG) and *in vitro* maturation (IVM) methods are influenced by the characteristics of the tissue, the culture environment and the stage of the oocyte at the start of culture. Many culture systems have been developed to culture follicles based on the needs of each species. To obtain the best results, culture medium, supplementation, temperature, concentration of oxygen and culture method have to be standardized.

The most common medium used is minimal essential medium (to cite a few studies, [43,44]) although other media like Waymouth [45] and McCoy's 5a have also been used [43,45,46]. Different medium supplements has been used to improve follicle development at culture, some examples are: antibiotics/antimycotics, ITS (insulin, transferrin and selenium), growth factors, gonadotrophins, activin or apoptosis inhibitors. Doses of gonadotrophins are critical for the proper progression of the culture. For instance, supplemented medium with follicle stimulant hormone (FSH) and LH are essential for the progression of the follicle from preantral stages to antral follicle stage [46–48].

The supplementation with serum is a topic of considerable debate. Serum contains multiple substances which could promote cell adhesion and proliferation. Serum acts as a source of albumin, which balances the osmolarity, and as a scavenger that protects from potentially harmful molecules like free oxygen radicals. Serum also acts as a source of precursors for steroid biosynthesis [49]. However, supplementation with serum has been related to a diminution of proliferation rates [50,51]. Other important conditions are the media used for transportation and maintaining the tissue [52,53], culture medium pH [54], characteristics of the extracellular matrix [54,55] and the percentage of oxygen [56]. The efficacy of supplements depends on the culture system used but it is considered that FSH, insulin, activin A, growth and differentiation factor 9 (GDF9) promote follicular development and survival (reviewed by [57]).

The characteristics of the ovary vary with the species used; in other words, ovaries from mouse, cow, cattle, pig or human are anatomically and physiologically different. In mouse, follicles can be obtained from neonatal, pre-puberal or adult ovaries. Follicles from neonatal or pre-puberal ovaries permit a greater number of follicles to be obtained than adult ovaries. Moreover, obtaining the follicles from neonatal and pre-puberal ovaries also improves follicular culture because all follicles are at the same developmental stage, they are all primordial follicles [58,59].

Different follicle isolation methods (mechanical and enzymatic) have been developed to obtain pre-antral follicles to seed the cultures. The isolation method is chosen taking into account the characteristics of specie selected to be cultured. There are pros and cons associated with each isolation method. In general, mechanical isolation keeps the structure of the follicle intact but the number of follicles obtained could be lower than using enzymatic methods [46]. On the other hand, enzymatic methods permit a greater number of follicles but can induce cell damage which may compromise the subsequent culture [50,51,60].

Different protocols have been described to culture follicles, in summary we can separate them into those that culture ovarian pieces and those that culture isolated primordial follicles (reviewed by [48,57]). Ovarian tissue culture has been conducted for a number of species (mouse, cattle, baboon, monkey and human) over varying time of culture, culture media and supplements, but definitely the best result were obtained in mice [46,54,61–63].

The first live mouse pup from cultured follicles was described by Epigg and O'Brien [60] using a two steps protocol. Authors took mouse ovaries and cultivated them for eight days, after that they enzymatically disaggregated the ovary and obtained isolated preantral follicles. These preantral follicles were further cultivated for another 14 days. This method was successful in part because the characteristics of the development of the mouse make it possible: mouse follicles are formed a few days after birth and the numbers of follicles and the stage of development are very similar among all the follicles. In fact, nowadays the mouse is the only specie that allows the progression of primordial follicle to a competent oocyte *in vitro* (reviewed by [57]). Using this method, follicles may remain viable and morphologically normal for up to three weeks in culture. Furthermore, multilayer pre-antral follicles have been isolated following the culture of fragments of ovarian cortex [46] or after culture of mouse follicles and ovaries [45,60].

Telfer *et al.* [46] cultured stroma-free small human ovarian cortex slices containing only primordial follicles. After six days of culture in serum-free medium, they observed pre-antral follicles. After that, obtained pre-antral follicles were cultured for four days more in an Activin A supplemented medium. Follicles treated with Activin displayed normal morphology, intact oocytes and antral formation. Authors demonstrated that with an adequate supplement, human primordial follicles could progress to pre-antral follicles [45]

Many protocols have used isolated primordial follicles from multiple species to seed the culture (to mention some of the first ones in different species see pig [64], bovine [65] and humans [66,67]. In most culture conditions, a great number of primordial follicles are viable following extraction but after 24 h they rapidly lose their three dimensional structure: the follicles collapse, the pre-granulosa cells migrate away from the follicle and oocyte degenerate [43,68]. A more recent study described a reproducible two-step culture method for isolated mouse primordial follicles [69]. Isolated follicles were cultured for six days, avoiding the theca cell to attach to the culture surface. After these six days of culture, attachment of theca cells was allowed and follicles were cultured for another 12 days. Authors described an average meiotic maturation, as defined by oocytes being able to mature to metaphase II after appropriate hormonal stimulation. Nevertheless, estrogen secretion was lower than that obtained from pre-antral cultured follicles.

Finally, many efforts have been applied to culture pre-antral follicles either obtained from fresh tissues or from some of the culture techniques mentioned above. Pre-antral follicle culture methods can be divided into those that allow follicle attachment, thus losing the architecture of the follicle [43,44] or those culture systems that maintain tridimensional follicle integrity. Three dimensional methods (3D) maintain the architecture of the follicle and they usually use serum-free medium [46,51,62], hydrophobic membranes [70] or a collagen matrix [71,72] or alginate gels to encapsulate the follicles [73,74]. In general, all these methods allow rodents follicles to reach mid-pre-antral sized follicles after six days. Rodent follicles cultured with 3D methods have apparently normal morphology and steroid production [68–70], respond to exogenous ovulatory stimulus [75] and the obtained oocytes could be fertilized [68].

Pre-antral follicle culture has also permitted the successful *in vitro* development of follicles obtained from either fresh [43] and cryopreserved tissues [76–78]. Follicles obtained from cultures that used an attachment IVG method, showed an oocyte diameter, chromatin configuration, transcriptional activity and meiotic competence similar to those seen *in vivo* [79]. 3D IVG methods originated for the culture of mouse pre-antral follicles [43,50,68,70,71], but also could be applied to follicles from domestic ruminants and human. In these species, the use of extracellular matrices to support the architecture of follicles in inert alginate hydrogels has been shown to be a good option. The alginate matrix mimics the stromal microenvironment of the ovary and is a good support to the growth and maturation of multilayered secondary follicles in pigs, cattle, ruminants, primates and humans [76,80–86]. Early reports demonstrated that murine follicles grown in alginate capsules reached the sizes observed *in vivo* [81,87]. Extended growth, coordinated differentiation of follicular cell types, antral cavity formation, theca cell differentiation, oocyte maturation and hormone production were described in 3D methods for mouse, monkey and human (reviewed by [57]). Nonetheless, the quality of the oocytes derived from follicles with accelerated growth rates needs to be fully verified, especially before these systems can be considered for clinical use.

Nevertheless, in most of these reports, meiotic progression is not directly evaluated and the progression in culture is related to the specie. Only in mouse is it possible to obtain a live pup from a primordial follicle, in the other cases the outcome depends on the stage of the follicle at the start of the culture.

## 6. Conclusions

The study of mammalian meiosis is a crucial topic to understand the basis of human infertility and aneuploidy origin. However, due to the shortage of samples and the impossibility to manipulate human gonads, studies in humans are scarce. Therefore, most knowledge obtained until today comes from the study of model organisms, like mouse, that permit genetic manipulations. Nevertheless, as discussed before, generating genetically engineered mice involves time and substantial costs to create and maintain the line. Thus, achieving a culture technique that would allow meiotic progression *in vitro* could be a very useful tool to study mammalian meiosis. Not only would it permit the manipulation of the human meiotic process and thus to test hypotheses that for the moment cannot be tested, but also it could complement the *in vivo* studies performed in mutant mice. For instance, a meiotic culture could enable the study of synapsis progression live under the microscope. Also, a synchronic culture could be a very powerful tool to study certain meitoic processes from a biochemical point of view, to mention some of the multiple possibilities that such a technique could offer. Meiotic culturing is already being used to test the genotoxicity of physical and chemical agents on spermatogenesis [29,34] or oogenesis [88].

Some of the results obtained to date are promising and, although most culture techniques are far from being a ready-to-use protocol for many labs, it is exciting to see a future in which protocols to culture mammalian sperm or oocytes precursor cells will be a common and useful instrument for the study of gametogenesis and meiosis.
